# Stromal corneal dystrophy (possible Schnyder’s dystrophy) with peripheral corneal degeneration – diagnostic and therapeutic challenges

**Published:** 2018

**Authors:** Mihail Zemba, Raluca Neacsa, Bogdan Ion Cucu

**Affiliations:** * Department of Ophthalmology, “Dr. Carol Davila” Central Military Emergency University Hospital, Bucharest, Romania; ** ”Carol Davila” University of Medicine and Pharmacy, Bucharest, Romania; *** Laser Vision Med, Constanta, Romania

**Keywords:** Schnyder’s crystalline dystrophy, peripheral corneal degeneration

## Abstract

**Purpose:** to highlight the diagnostic and therapeutic challenges in a case with central and peripheral corneal lesions.

**Methods:** We present the preoperative investigations: anterior segment optical coherence tomography and ultrasound biomicroscopy;

The excised cornea has been examined histopathologically.

**Results:** Preoperative assessment showed that peripheral lesion was not ectatic;

Histopathological examination points to a possible corneal degeneration, with posttraumatic or infectious etiology for the peripheral lesion and gives some reasons to sustain the diagnosis of Schnyder’s dystrophy for the central lesion.

**Conclusions:** The association of different corneal lesions may need many investigations to establish the diagnosis and choose the most appropriate therapeutic solution.

We present the case of a 48-year-old male patient, referred to our clinic for bilateral decreased visual acuity, more pronounced on right eye.

History of the eye disease started at the age of 15; then the visual acuity decreased; at the age of 20 the patient got his driving license; the visual acuity was the same in both eyes, but he was not able to distinguish the very small rows on the eye chart. Eight years before he described an incident when the right eye was very red and painful, however, the visual acuity did not modify significantly at that time. The treatment consisted in different eye drops, but the patient had no medical records and could not specify the treatment. He noticed a decrease in visual acuity of both eyes in the last year, more pronounced on the right eye.

**Ophthalmological examination:**

Visual acuity:

O.D. – counts fingers at 1 feet

O.S. – 0,1

Refraction – cannot be performed

Intraocular pressure:

O.D. – 14 mm Hg.

O.S. – 13 mm Hg.

Biomicroscopy:

O.D.

The corneal epithelium was intact, fluorescein staining showed no lesion. A discoid corneal opacity was located centrally, predominantly in the anterior half of the stroma, with polychromatic crystals, particularly in the centre of the lesion. The diameter of the lesion was around 5 mm. The cornea surrounding the lesion was clearly 2 millimeters from the lesion, except for the upper side, where the transparency was only for 1 millimeter. A corneal arcus was noticed nasally, temporally and inferiorly. 0,5 millimeter of clear cornea remained near the limbus.

Another lesion was noticed superiorly, between 10 and 14 o’clock. It started from the limbus to the centre of the cornea. The overlying epithelium was intact. Cornea seemed thinner than normal and it was opaque in all layers. Corneal neovascularization started from the limbus to the edge of the lesion, that was located approximately 3 millimeters from the limbus (**Fig. 1**).

**Fig. 1:**
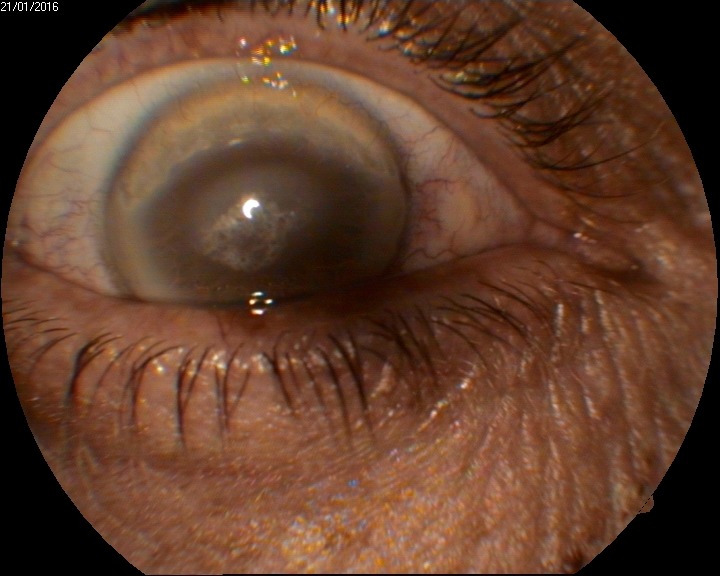
Biomicroscopy O.D.

O.S.

A discoid opacity was located in the centre of the cornea. The dimension was the same as in the right eye, but the polychromatic crystals were less obvious. The surrounding cornea was clear. There was a corneal arcus in the periphery, on the entire corneal circumference (**Fig. 2**).

**Fig. 2:**
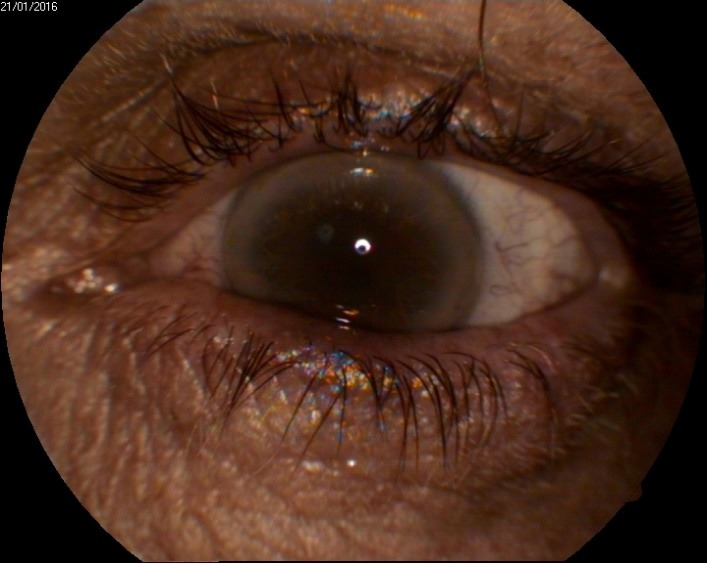
Biomicroscopy O.S.

**Presumptive diagnosis was:**

O.D. Stromal corneal dystrophy. Superior corneal degeneration.

O.S. Stromal corneal dystrophy.

Ultrasound biomicroscopy was not very helpful. There was a central corneal hyperreflectivity, with an almost equal intensity in all layers and the peripheral stroma was less reflective. This might have been related to the central stromal opacity, but in ultrasound biomicroscopy images, cornea was more reflective in its central part, so that aspect could be normal (**Fig. 3**).

**Fig. 3:**
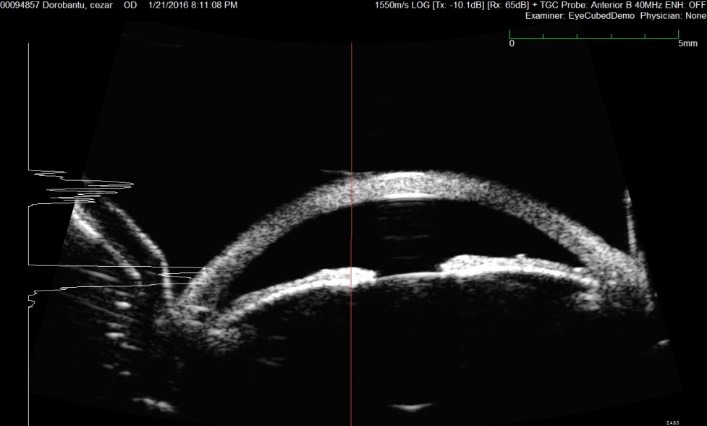
Ultrasound biomicroscopy O.D. – Central hyperreflectivity

What was more important, the peripheral cornea did not show a significant thinning in the area of degeneration; on the contrary, the thickness was higher in that specific zone than in the centre of the cornea – as in a normal cornea (**Fig. 4**).

**Fig. 4:**
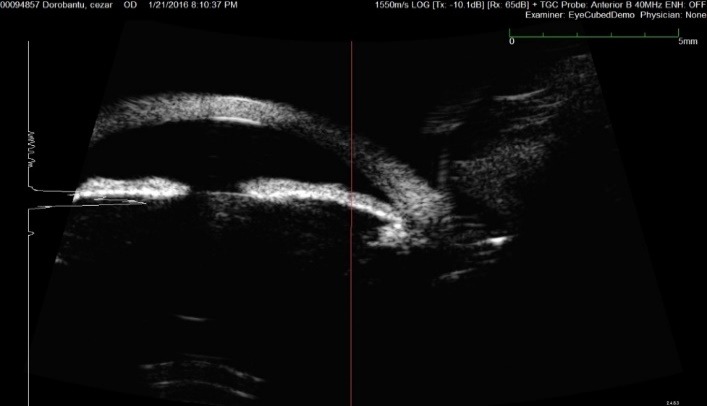
Ultrasound biomicroscopy O.S.

Optical coherence tomography of the anterior segment was more useful. A section at the level of central lesion showed a normal epithelium and a central hyperreflectivity, more pronounced in the anterior half of the stroma (**Fig. 5**).

**Fig. 5:**
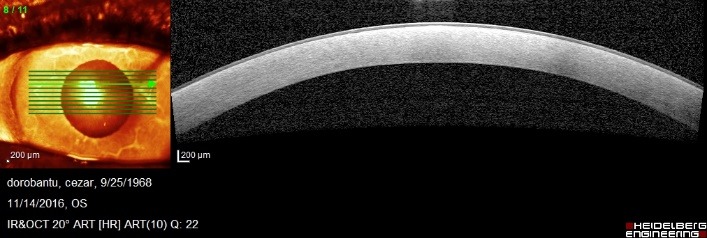
Optical coherence tomography O.D. – section at the level of central lesion

Another section, realized at the level of the clear cornea between the central and peripheral lesion showed a normal cornea in the centre of the image and some changes in the periphery of the image, where the section crossed through the peripheral lesion. Fortunately, the thickness of peripheral cornea did not seem diminished (**Fig. 6**).

**Fig. 6:**
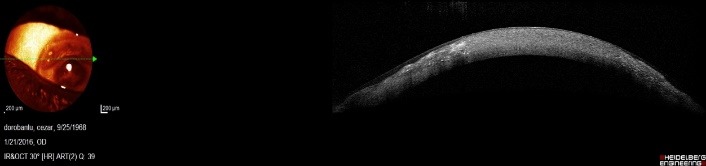
Optical coherence tomography O.D. – section at the level of clear cornea between central and peripheral lesion

The last section, at the level of peripheral lesion, showed a normal epithelium above the lesion. The structure of the cornea alternated reflective areas with less reflective areas in a random distribution. The thickness of the peripheral cornea was normal (**Fig. 7**).

**Fig. 7:**
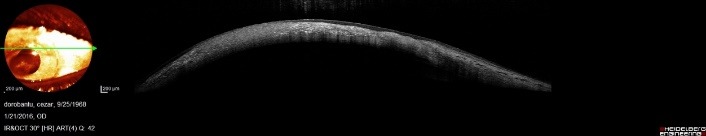
Optical coherence tomography O.D. – section at the level of peripheral lesion

**Laboratory findings:**

Cell blood count with white blood cell count - normal

- glucose – 98 mg/ dl

- cholesterol – 340 mg/ ml

 - triglycerides – normal values

 - total lipid profile – normal values

- serum protein electrophoresis- normal values

 - immunogram-normal values

 - rheumatoid factor - negative

Only serum cholesterol had a higher value, other laboratory tests were in normal range.

**Positive diagnosis was:**

O.D. Stromal corneal dystrophy (possible Schnyder’s type) Peripheral corneal degeneration

O.S. Stromal corneal dystrophy (possible Schnyder’s type)

**Differential diagnosis**

**For the central lesion:**

1.Bietti crystalline corneal dystrophy has lesions predominantly in the perilimbal peripheral stroma. Moreover, there are peripheral retinal changes with impaired night vision (absent in our patient).

2.Corneal deposits from severe dysproteinemia, multiple myeloma, Waldenstrom’s macroglobulinemia, benign monoclonal gammopathy – there are no laboratory findings related to these diseases.

3.Other stromal dystrophies: granular corneal dystrophy type I Groenouw, macular corneal dystrophy type II Groenouw and lattice dystrophy show different biomicroscopic changes in cornea.

**For the peripheral lesion**

1.autoimmune diseases ulcers: rheumatoid arthritis, polyarteritis nodosa – there are no clinical and laboratory findings for these diseases.

2.marginal keratitis – a clear area remains between the ulcer and the limbus.

3.phlyctenulosis – less extensive in surface.

4.Mooren’s ulcer – is characterized by important thinning of the cornea.

5.pellucid marginal degeneration – is located most frequently inferiorly.

6.Terrien’s marginal degeneration – is located superiorly, as in our patient, but it induces a significant astigmatism and is bilateral, although there are asymmetrical cases.

**Treatment**

The patient underwent a penetrating keratoplasty. The surgery had a normal evolution, with no intraoperative incidents.

Postoperative assessment for days after surgery: visual acuity was 0,2, clear donor cornea, clear lens, fundus examination with normal appearance.

Three months after surgery, the visual acuity was 0,4, with clear donor cornea (**Fig. 8**).

**Fig. 8:**
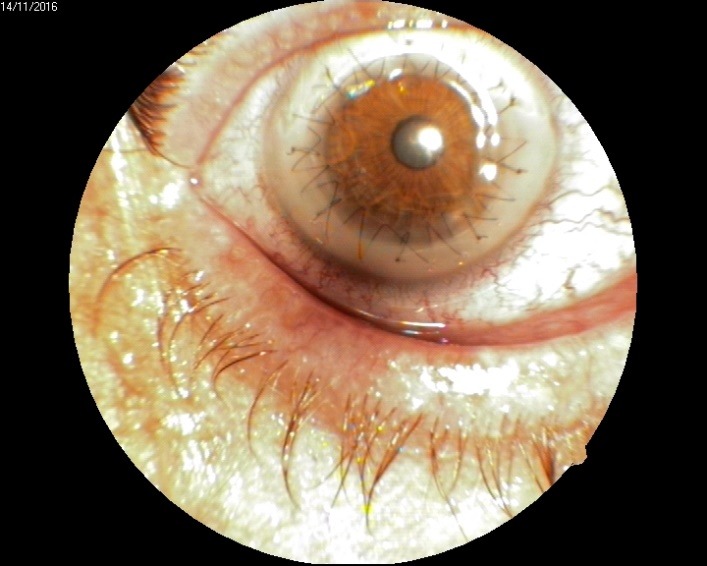
Anterior pole day 4 after penetrating keratoplasty

Histopathologic examination of the excised cornea showed some findings well related with Schnyder’s dystrophy:

- Lipid deposits in the stroma at different levels, predominant anterior, but also in posterior stroma (**Fig. 9**).

**Fig. 9:**
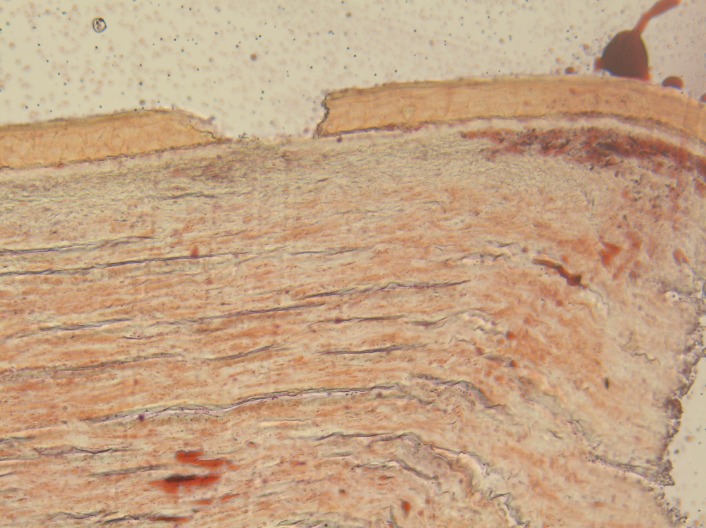
Red oil O staining; 40x magnification – lipid deposits in anterior and posterior stroma

- Lipid accumulation throughout the stroma (**Fig. 10**)

**Fig. 10:**
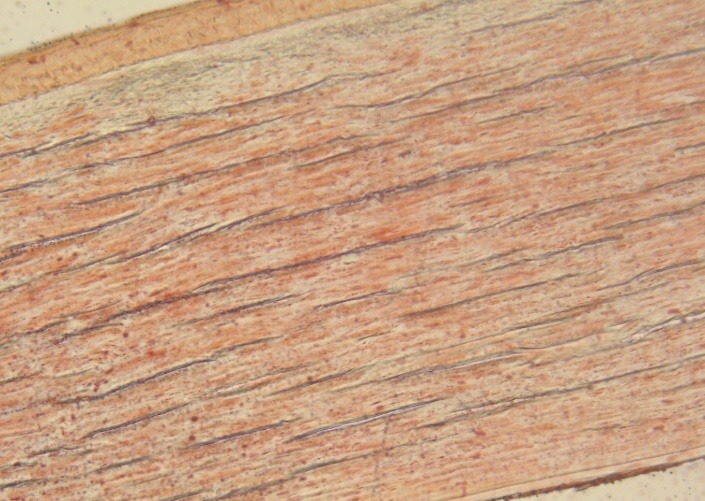
Red oil O staining; 40x magnification – lipid deposits throughout the stroma

**Fig. 11** is the control color witness (10x magnification)

**Fig. 11:**
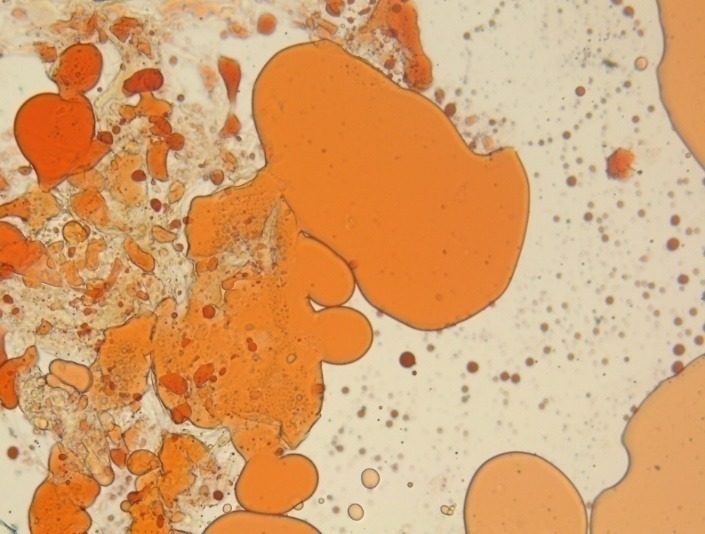
Control color witness (10x magnification)

The histologic examination of the peripheral lesion showed a normal structure of the epithelium; at the level of the stroma, there were macrophages with intense colored nuclei, hyaline transformation in the anterior part of the stroma and small areas of neovascularization. In the left part of the image, there was a normal aspect of the cornea (limit of the peripheral lesion). The aspect was compatible with an inflammatory reaction that was healed or almost healed – macrophages may be a sigh of a chronic low level inflammation in that region (**Fig. 12**,**13**).

**Fig. 12:**
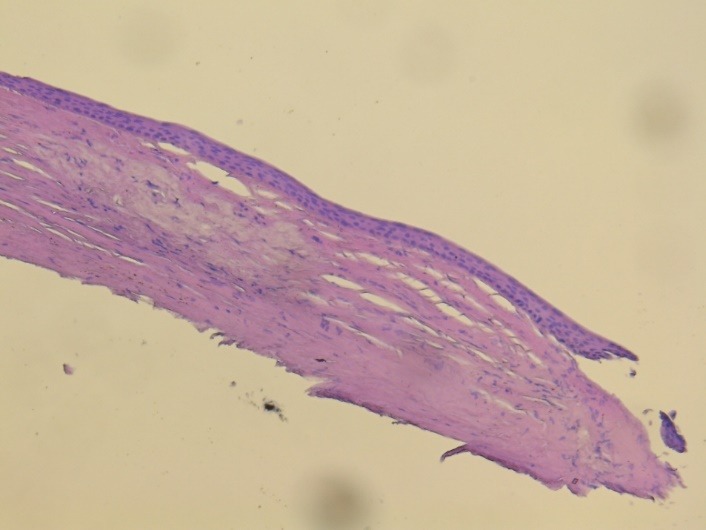
– hematoxylin – eosin staining; 5x magnification

**Fig. 13:**
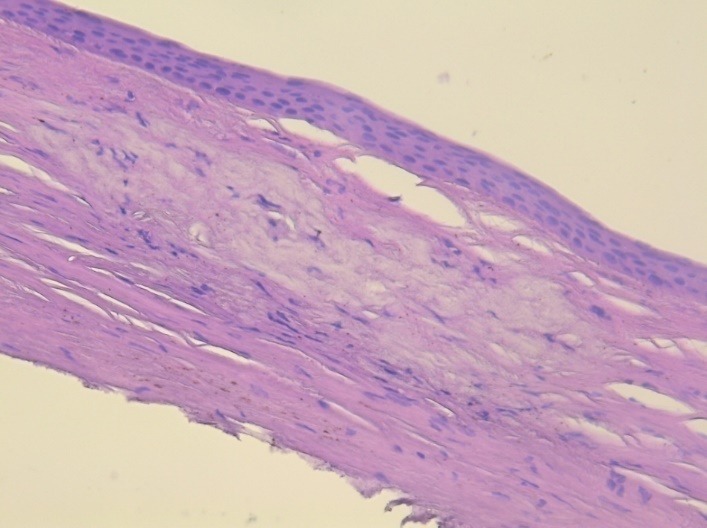
hematoxylin – eosin staining; 10x magnification

**Prognosis**

The short and medium term prognosis was good. One year after surgery the patient had a good visual acuity – 0,5, with clear cornea. The long-term prognosis can be affected by the relapse of the dystrophy in the donor cornea.

## Discussions

The Schnyder’s crystalline dystrophy was described for the first time by Wibaut and Van Went in members of three generations of a family [**1**]. The main characteristics of this dystrophy were described later by Schnyder [**2**].

In the IC3D classification, this dystrophy is a category 1 (C1) dystrophy: a well-defined corneal dystrophy in which the gene has been mapped and identified and the mutations are known [**3**]. The inheritance is autosomal dominant, related with gene UbiA prenyltransferase domain, at the genetic locus 1p36 [**4**].

The diagnosis of Schnyder’s crystalline dystrophy in our patient was based on clinical signs: bilateral, disk-like lesion, central location, with decreased visual acuity since the age of 15, polychromatic crystals, arcus senilis at the age of 48, but also on histopathologic findings: lipid, neutral fat and cholesterol deposits at all levels of the corneal stroma.

The pathologist needed to be informed that we wanted to identify lipids and cholesterol in the cornea specimen, because the preparation of the piece was different. Organic solvents and resins routinely used for examining the pieces can dissolve most of the fats, so that special lipid stain, as oil red O or Sudan black, are necessary to identify the lipids in the cornea [**5**,**6**].

There were also some problems related to the surgery. The suture was performed in the region of the upper peripheral degeneration. We did not know the exact nature of that lesion. It seemed to be cicatricial, non-active, but it was difficult to foresee the outcome, the reaction of that area to the sutures (irritation, inflammatory reaction, rejection). Another important factor difficult to assess was postoperative astigmatism, which could be induced by a suture in that area.

## Conclusion 

The association of different corneal lesions may need many investigations to establish the diagnosis and choose the most appropriate therapeutic solution. In our case, we found out that the thickness of the cornea was quite normal in the area of the peripheral degeneration, so we performed a penetrating keratoplasty with a very good result.
